# Privileged Scaffolds for Potent and Specific Inhibitors of Mono-ADP-Ribosylating PARPs

**DOI:** 10.3390/molecules28155849

**Published:** 2023-08-03

**Authors:** Maria Giulia Nizi, Chiara Sarnari, Oriana Tabarrini

**Affiliations:** Department of Pharmaceutical Sciences, University of Perugia, 06123 Perugia, Italy; chiarasarnari97@outlook.it

**Keywords:** PARPs, PARPs inhibitors (PARPi), mono-ARTDs, selective inhibitors, chemical probes

## Abstract

The identification of new targets to address unmet medical needs, better in a personalized way, is an urgent necessity. The introduction of PARP1 inhibitors into therapy, almost ten years ago, has represented a step forward this need being an innovate cancer treatment through a precision medicine approach. The PARP family consists of 17 members of which PARP1 that works by poly-ADP ribosylating the substrate is the sole enzyme so far exploited as therapeutic target. Most of the other members are mono-ADP-ribosylating (mono-ARTs) enzymes, and recent studies have deciphered their pathophysiological roles which appear to be very extensive with various potential therapeutic applications. In parallel, a handful of mono-ARTs inhibitors emerged that have been collected in a perspective on 2022. After that, additional very interesting compounds were identified highlighting the hot-topic nature of this research field and prompting an update. From the present review, where we have reported only mono-ARTs inhibitors endowed with the appropriate profile of pharmacological tools or drug candidate, four privileged scaffolds clearly stood out that constitute the basis for further drug discovery campaigns.

## 1. Introduction

Post-translational modifications (PTMs) are a series of chemical reactions involving the attachment of different groups into a target protein. More than 400 different PTMs have been identified able to affect protein structure, functions and working as essential regulators of cellular processes. Being these modifications involved in signal transduction, gene expression regulation, gene activation, DNA repair and cell cycle control, their disruption or alteration is responsible for cellular dysfunction and various pathologies [1].

Among the most common PTMs, such as phosphorylation, ubiquitylation, acetylation, or methylation, ADP-ribosylation plays essential roles both in prokaryotic [2] and eukaryotic cells [3]. It is catalyzed by ADP-ribosyltransferases (ARTs) and, in mammalian cells, most of them belong to the class of diphtheria-toxin-like ADP-ribosyltransferases (ARTDs) which comprises poly-ADP-ribose polymerases (PARPs) and tankyrases (TNKS) [4,5].

**Figure 1:**
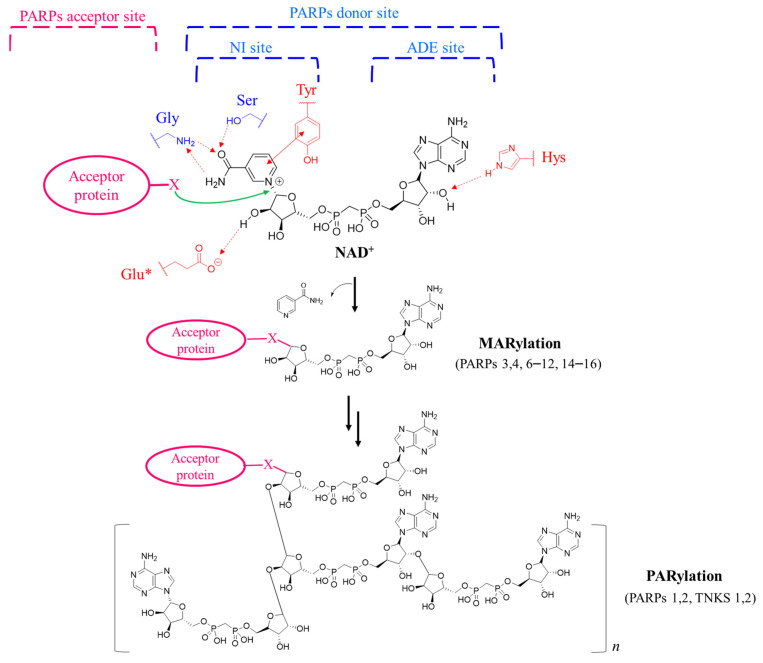
Schematic representation of PARPs catalytic domain and mono-/poly-ADP-ribosylation of target protein mediated by PARPs using NAD^+^ as co-substrate. The key interactions of NAD^+^ with the PARPs donor site are also shown. The amino acids marked in red belong to the catalytic triad, *: Glu is replaced by hydrophobic residue in mono-ARTs. X = acceptor amino acid of the target protein.

There are 17 PARP enzymes, they share a common catalytic domain able to bind the substrate *β*-nicotinamide adenine dinucleotide (NAD^+^) and transfer ADP-ribose units of NAD^+^ to acceptor nucleophilic amino acids residues of target proteins, with the nicotinamide portion that is released as a by-product (Figure 1). Although proteins represent the principal targets of PARPs, these enzymes also modify nucleic acids, both phosphorylated DNA and RNA ends, representing another physiologically relevant form of reversible ADP-ribosylation signaling [6,7].

Based on the PARP catalytic activity, the ADP-ribose can be attached to the target protein in form of monomer (MARylation) or polymers (PARylation). The poly-ADP-ribosylation is catalyzed by four out 17 PARP enzymes, PARP1 and 2 and TNKS1 and 2 (referred to as poly-ARTs) [8]. The polymers can be characterized up to 200 units of ADP-ribose linear or branched every 20–30 units; however, oligomers from 2 to 20 units are more widespread. With the exception of PARP13 that is inactive, all the other enzymes catalyse the mono-ADP-ribosylation (mono-ARTs), with PARP9 that is reported to be able to MARylate ubiquitin only when complexed with an E3 ubiquitin ligase [9].

The differences of the catalytic activity derive from variances in the catalytic triad of amino acids: the first position of the triad is occupied by a histidine (H) both in mono- and poly-ARTs, while glutamine and tyrosine replace histidine in PARP9 and PARP13, respectively [10]. The second amino acid is represented by tyrosine (Y) in all the PARP subfamilies. This H-Y motif is essential for the correct accommodation of NAD^+^ and, as demonstrated by the inactive members, the loss of even one of them prevents the catalytic activity. Indeed, the conserved histidine forms a H-bond with the 2-OH of the adenosine ribose, while the tyrosine generates a π-π stacking with the nicotinamide moiety (Figure 1) [10]. In the third position of the triad, a glutamate (E) residue characterizes the poly-ARTs (H-Y-E) and it is involved in the elongation of the ADP-ribose chain. Glutamate is replaced by a hydrophobic residue, such as leucine or isoleucine in the mono-ARTs (H-Y-Φ), thus preventing the polymerase activity [11].

In the PARPs catalytic domain, two main sub-sites can be recognised: donor site and acceptor site (Figure 1), surrounded by donor (D) and acceptor loops, respectively. The donor site binds NAD^+^ and is composed of a nicotinamide (NI) site, phosphate binding site and adenine ribose (ADE) site. In the NI site, the most important interactions are generated by π-π interactions between highly conserved tyrosine residues and nicotinamide ring and by three H-bonds between serine and glycine residues and the amide group of nicotinamide. In the acceptor site, instead, the enzyme interacts with the macromolecule to be modified and it is also the site in which the elongation of ADP-ribose chains occurs. 

Similar to other transient biological processes, the ADP-ribosylation relies on synthesis and degradation mechanisms; indeed, ADP-ribosylation is reversed by various “eraser” involved in the degradation at the various points of the chain: PARP glycohydrolase (PARG) turns the PARylation into MARylation, while other enzymes, such as TARG, ADP-ribosylhydrolase 3 (ARH3), or macrodomains, depending on the type of acceptor amino acid, cleave the ADP-ribose-aminoacid linkage [12,13,14].

The first member of the family, PARP1, was discovered in 1963 and plays an important role in the detection and repair of DNA single-strand breaks (SSBs) [15]. In particular, the DNA damage is rapidly detected by PARP1 and their interaction allows the PARylation of multiple proteins, including chromatin-associated proteins [16], which are essential for the activation of the DNA damage repair machinery. PARP1 is also able to auto-modify itself, determining a conformational modification responsible for its detachment from the DNA helix. PARP2 and PARP3 also play key roles in the DNA damage repair with some common functions and redundancy in structure, particularly between PARP1 and PARP2 [17].

The roles of these enzymes in the DNA repair made them drug targets of special interest. In cancer cell lines with defective breast cancer genes 1 and 2 (BRCA1/2), the PARP’s roles emerged as even more essential for the survival [18,19]. Indeed, by inhibiting these enzymes, a selective cell death of the diseased cells is achieved based on a synthetic lethality [20], a phenomenon in which the occurrence of a single genetic event is tolerable for cell survival, whereas the co-occurrence of multiple genetic events results in cell death [21]. Thus, in 2014, the first PARP inhibitor (PARPi), olaparib [22], was approved by FDA and EMA, followed by three other congeners: rucaparib (2016) [23], niraparib (2017) [24] and talazoparib (2018) [25]. In addition, veliparib [26] is approved for use under an orphan designation, and it is in phase III clinical trials, while the last drug entered the clinical trial is saruparib, the first really selective PARP1 inhibitor, which is currently in phase I/II of clinical trials. 

The approved drugs are used to treat cancers such as ovarian, breast, peritoneal, or prostate with BRCA1/2 mutations, which for breast and ovarian cancers account for 10–15% frequency [18,27,28]. Recent studies have shown that the susceptibility to PARPi treatment can also go beyond the BRCA1/2 mutation, suggesting that other mechanisms of action can contribute to their anticancer effects, including the so-called “trapping” effect [19,29,30]. 

The other poly-ARTs, TNKS1/2, which play a key role in the Wnt/β-catenin signaling control [31,32], have been also largely explored as anticancer targets and, although no inhibitors are on the market, many TNKS inhibitors continue to appear in the literature [33,34], with the selective TNKS inhibitor STP1002 [35] and the dual TNKS/PARP1-2 inhibitor E7449 (stenoparib) [36] that are in phase I and II, respectively, of the clinical trials. 

Respect to poly-ARTs, the mono-ARTs are characterized to a lesser extent, with an interest that has increased in recent years highlighting important biological roles, as mentioned below, that elect them as very promising targets for future drug design. Most important, their inhibition let to foresee a wider therapeutic applications respect to poly-ARTs, covering also non-oncological disorders such as inflammation and neurological diseases [37].

About 10 years ago the first small molecules inhibitors of mono-ARTs appeared in the literature, and over time, a handful of compounds expanded their chemical space, with two of them that entered clinical trials as anticancer or anti-inflammatory. However, there is still an urgent need to dispose of proper chemical tools for interrogating the biological roles of these underexplored enzymes and to expand the range of druggable targets. Since the first perspective on mono-ARTs inhibitors, published in early 2022 [38], five additional compounds have been disclosed that, for their potency and selectivity, represent a precious step forward in the mono-ARTs field. This prompted us to prepare the present review where we have analyzed these compounds along with the previous most promising mono-ARTs inhibitors, highlighting as only a few privileged scaffolds are able to furnish compounds with the desired profile of chemical probe or clinical candidate. 

The inhibitors herein reported should inspire the medicinal chemist’s community to further invest on this field in order to identify even more potent and selective inhibitors that could culminate in innovative drugs.

### Mono-ARTs Functions

**Figure 2:**
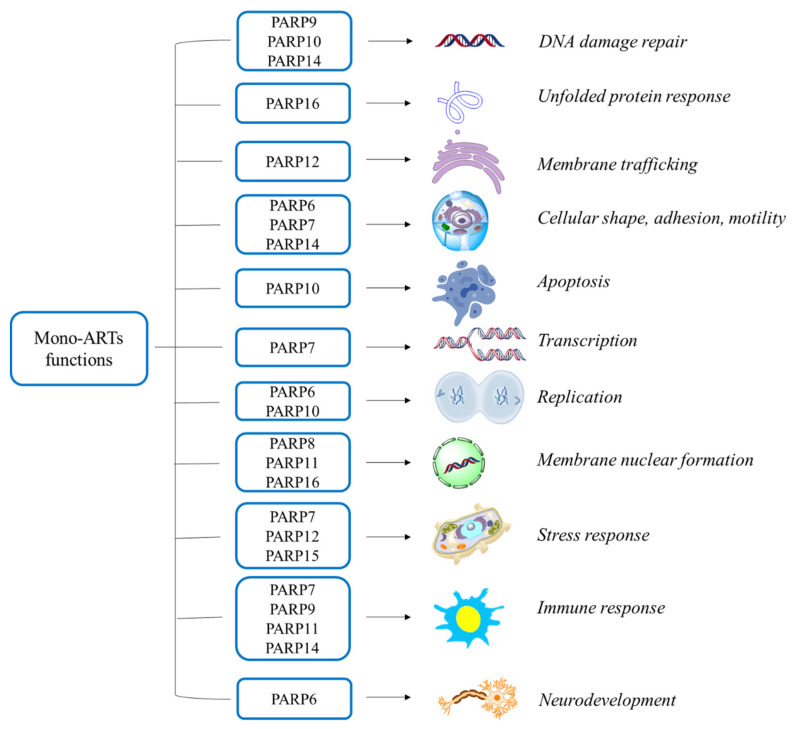
Key functions played by mono-ART enzymes.

Mono-ARTs have gained the attention of the scientific community in 2008 when PARP10 was identified as the first mono-ART member. Since then, the consideration has been growing and they emerged as therapeutically powerful enzymes with important roles in many key processes such as the regulation of apoptosis and cell replication, the stress and immune response modulation or the neurodevelopment (Figure 2). 

PARP10 plays disparate roles: cooperating with the proto-oncoprotein c-Myc, it regulates the cell cycle and apoptosis [39]; while its interaction with PCNA is essential for the restart of the replication fork in the presence of DNA damage [40]. In addition, being that PARP10 is involved in the MARylation of some kinases, including the mitotic kinase Aurora-A, it was recently demonstrated that PARP10 depletion leads to defects on the G2/M phase transition [41]. Finally, PARP10 is also involved in Wnt signaling, in which Gsk3β has been identified as one of its targets, and, thus, it has been linked to immune regulation and metabolism [42]. From a pathological point of view, it was shown that PARP10 supports cell growth during the late G1-S cell cycle phase and improves the DNA damage tolerance during S phase [43]. Of note, a potential role of PARP10 in inflammation can also be envisioned: as a part of NF-kB signaling, it inhibits the nuclear translocation of the transcription factor p65 thus reducing gene expression [44]. 

PARP14 is the most studied member with many roles already described. It is a well-known transcription co-factor in macrophages where it suppresses the IFN-γ response [45,46] and it is able to regulate STAT6 and IL-4-mediated transcription [47]; PARP14 is also involved in various signal transduction pathways, including the NF-kB pathway with a stimulatory effect [48] and the JNK1-JNK2 axes with an inhibitory activity [49], and, finally, it also regulates mRNA stability. More than 100 proteins have been identified as PARP14 targets, which are involved in translation, DNA repair or metabolism [50]. For all the above essential roles, a PARP14 dysregulation has been already linked to a series of pathologies. Concerning its involvement in cancer disease (recently reviewed [51]), it was found that PARP14 is overexpressed in multiple solid and liquid tumors such as large B cell lymphoma [52], multiple myeloma [49], prostate cancer [53] and hepatocellular carcinoma [54] (Table 1). In particular, MARylation of histone deacetylase (HDAC) 1 and 2 by PARP14, in turn activated by the IL4-STAT6 pathway, promotes the activation of factors essential for gene transcription. In addition, PARP14 overexpression, mediated by JNK2, negatively regulates JNK1-dependent apoptosis in the 80% of multiple myeloma [49]. Moving to inflammation, by amplifying STAT6- and STAT3-driven transcription [45,47], PARP14 is involved in Th2/Th17 signaling in immune response [55], and elevated PARP14 expression is found in tissues derived from patients with idiopathic pulmonary fibrosis, atopic dermatitis and psoriasis, as well as in epithelial and inflammatory cells. 

Another mono-ART for which numerous biological roles have emerged is PARP7. It is expressed in multiple cell and tissue types. It is a key effector of signaling and gene expression, with regulatory roles in androgen receptor, estrogen receptor and liver X receptor transcription [56,57,58]. PARP7 is also involved in the negative regulation of kinase TBK1 [58]. In particular, PARP7 was proposed to reduce the sensitivity of the INF-I mediated response to cytosolic nucleic acids. The latter activity is also related to the PARP7 involvement in tumor progression. Indeed, the typical genomic instability that characterizes cancer cells could lead to the releasement of nucleic acids in the cytosol. Through a nucleic acid–sensing mechanism, the cell activates cGAS-STING and RIG-I, resulting in cell death or immune recognition mediated by the IFN-I pathway [59]. In some kind of cancer cells, however, no IFN-I activation can be detected. This is mainly caused by the mono-ADP ribosylation of TBK-1 mediated by PARP7, which prevents IFN-I activation [60]. For this reason, PARP7 inhibition could represent a valid anticancer strategy based on immunomodulatory effects through IFN-I response restoration that can allow the immune recognition of cancer cells and reverse immunoevasion [61]. In this way, the tumor cell progression is directly influenced and, indirectly, antitumor immune cells are also modulated [62]. Besides the immunomodulatory effect, it was also suggested that PARP7 could play a role in ovarian cancer growth and invasion by MARylating α-tubulin, thus promoting the microtubule instability [63]. On the contrary, it was also found that PARP7 works as negative regulator of estrogen receptor α (ERα) in MCF-7 cells, highlighting its role in the control and suppression of human breast cancer [64].

PARP12 is a mono-ART localized at the trans-Golgi network that possesses antiviral functions and is involved in the oxidative stress response. In particular, it exerts the antiviral activity, mainly against Zika [65], Chikungunya [66] and SARS-CoV-2 [67] viruses, by inhibiting the cellular RNA translation and by promoting the proteasome degradation of viral proteins, as also demonstrated by its overexpression upon viral infections [68]. In addition, under stress conditions, such as oxidative stress, heat or osmic shock, PARP12 moves from the Golgi complex to the stress granules, thereby promoting the stress response [69,70,71]. PARP12’s activity has been linked also to cancer diseases (Table 1), even if the role still remains elusive and controversial. Indeed, it seems to work as tumour suppressor in hepatocellular carcinoma metastasis [72] but, in contrast, PARP12 is upregulated in MCF7 breast cancer [73] where it is involved in resistance to chemotherapy. 

For all the other mono-ARTs, the physiopathology remains largely undefined with only a few data reported for PARP6, PARP11, PARP15 and PARP16. PARP6 regulates the G2-M phase cell cycle progression [74,75], and was also linked to the neurodevelopment, from late embryonic to the early postnatal stage, as demonstrated by its high levels in the hippocampus region of the brain [76]. For PARP11, an involvement in the IFN-I mediated response is reported [58,77]. PARP15 and PARP16 regulate the stress response, the former by promoting the stress granule formation [69,70], while PARP16 is involved in the unfolded protein response.

About their pathological roles, particularly in cancer diseases (Table 1), PARP6 contributes to the maintenance of centrosome integrity by MARylating the checkpoint kinase and promoting the breast cancer growth [78], while PARP15 is overexpressed in B-aggressive lymphoma [79] and acute myeloid leukemia [80]. They are also involved in non-oncological diseases, especially inflammation and allergic diseases, with a major involvement of PARPs 11, 15 and 16 that critically participate to the innate immune response regulations by inducing inflammatory cytokines such as IL-1, IL-6 or TNF-α and other pro-inflammatory mediators [60].

For a comprehensive overview of the mono-ARTs functions, the readers are directed to the following reviews [60,81,82,83,84,85].

**Table 1:** Mono-ARTs and their involvement in cancer disease.

Mono-ART	Cancer Type
PARP6	MDA-MB468 and HCC1806 breast cancers ^a^
PARP7	Advanced metastatic solid tumours ^b^
PARP10	Ovarian cancer ^c^ Breast cancer ^d^
PARP12	MCF7 breast cancer ^e^
PARP14	Large B cell lymphoma ^f^ Multiple myeloma ^g^ Prostate cancer ^h^ Hepatocellular carcinoma ^i^
PARP15	B-aggressive lymphoma ^j^ Myeloid leukaemia ^k^

^a^ Ref. [78]; ^b^ Ref. [61]; ^c^ Ref. [41]; ^d^ Ref. [86]; ^e^ Ref. [73]; ^f^ Ref. [52]; ^g^ Ref. [49]; ^h^ Ref. [53];^i^ Ref. [54]; ^j^ Ref. [79]; ^k^ Ref. [80].

## 2. Specific Mono-ARTs Inhibitors

The many physiopathological roles that continuously emerge for mono-ARTs, along with the possibility to exploit them as drug targets for disparate diseases, definitely ask for the identification of specific inhibitors, that, in parallel, represent precious tools to fully dissect their biology.

The number of mono-ARTs inhibitors is still very scarce with most of them that recognize more than one subfamily of PARPs [38]. However, a handful of inhibitors endowed with a high grade of selectivity for a mono-ART subfamily, coupled with nM potency, has been identified, especially in the very recent time, and they are the focus of the present paper. From this medicinal chemistry review, four nitrogen-rich heterocycles emerged as particularly privileged for obtaining potent and selective mono-ART inhibitors. We have sorted them based on their chemical scaffold; when available, the identification strategy, the hit-to-lead optimization and SAR studies, have been also reported.

### 2.1. Phthalazin-1(2H)-one

Phthalazinone-based compounds have been reported for a variety of biological functions, with zopolrestat and azelastine that are in clinical use as antidiabetic and antihistaminic, respectively, and various compounds that work as kinase inhibitors [87], or hepatitis B virus capsid inhibitors [88].

Phthalazinone is a well-known scaffold also in the poly-ARTs inhibitors field, which already furnished many compounds such as olaparib along with fluzoparib [89], which is under clinical development. 

More recently, this scaffold was exploited also for the synthesis of various mono-ARTs inhibitors.

**Figure 3:**
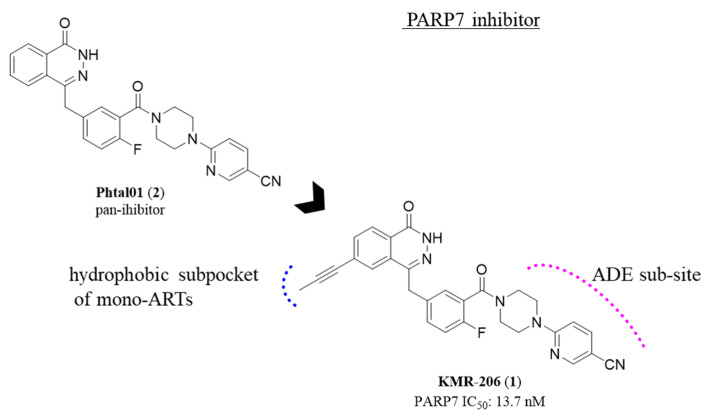
Structure and enzymatic activity of phthalazinone KMR-206.

In 2023 Cohen et al. [90] were able to identify a potent and specific inhibitor for the mono-ART PARP7, compound KMR-206 (**1**) (Figure 3). The authors started from the pan inhibitor Phthal01 (**2**) (Figure 3) [91], inhibiting PARP1 (IC_50_ = 21 nM), PARP2 (IC_50_ = 28 nM) and PARP7 (IC_50_ = 14 nM) and following the same approach applied in 2018 to obtain the specific PARP11 inhibitor ITK7 (see Figure 7 below) [92], a propynyl group was added at C-6 position, obtaining compound **1**. As expected, the hydrophobic substituent allowed to maintain the same potent activity of **2** against PARP7 with IC_50_ = 13.7 nM also reducing the affinity for poly-ARTs (PARP1 IC_50_ > 3 µM; PARP2 IC_50_ = 1 µM), with >200 and 75-fold selectivity for PARP7 over PARP1 and PARP2, respectively. Even if derivative **1** showed some activity also against PARP10 and PARP11 (IC_50_ = 179 and 134 nM, respectively), a good selectivity profile (>10-fold over PARP10 and 11) was maintained. In in-depth studies, compound 1 inhibited PARP7 auto-MARylation (in HEK293T cells) with IC_50_ = 8 nM, while decreased the cellular viability (in NCI-H1373 cell line) with a good EC_50_ = 104 nM, resulting however less potent of the PARP7 inhibitor RBN-2397 (EC_50_ = 17.8 nM, see Figure 6 below), which is under clinical development.

**Figure 4:**
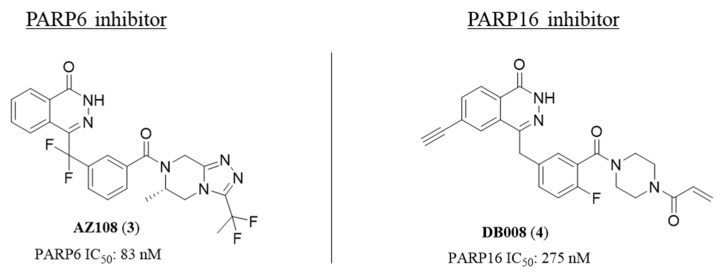
Structure and enzymatic activity of phthalazinones AZ108 and DB008.

A few other phthalazinones are worthy of mention due to their very potent activity against different mono-ARTs, with AZ108 (**3**) and DB008 (**4**) (Figure 4) that have been reported as the most potent inhibitors of PARP6 and PARP16, respectively, ever reported until now. In particular, AZ108 (**3**) was identified by AstraZeneca by assaying the in-house quinazolinone- and phthalazinone-based PARPi collection. It inhibited PARP6 with IC_50_ = 83 nM, but also PARP1 and PARP2 (IC_50_ = 30 nM) [74]. Although not specific, its anti-MDA-MB-468 breast cancer activity, in xenograft models, seems directly related to the PARP6 inhibition, which is responsible for blocking the centrosome clustering and inducing multipolar spindle.

DB008 (**4**) [93], (Figure 3) was identified more recently, in 2022, by Cohen et al., and was developed starting from olaparib that, besides the activity on PARP1 and 2, also inhibits PARP16 with IC_50_ = 3 µM. To achieve specific PARP16 inhibition, the authors replaced the terminal cyclopropyl moiety with an acrylamide in order to generate a covalent interaction with the Cyst169, uniquely present in PARP16 D-loop and added an ethynyl group at the C-6 position to reduce the affinity for poly-ARTs [92,94]. These modifications allowed to reach an IC_50_ value of 275 nM against PARP16 but, contrary to the expectation, the C-6 hydrophobic group was well tolerated by PARP2 that was inhibited even more potently (IC_50_ = 139 nM).

Compounds **1** and **4** demonstrated that the introduction of a hydrophobic substituent in C-6 position is not sufficient for mono-ARTs selectivity over poly-ARTs, differently from the SAR delineated for the quinazolinone scaffold (see below). This could be related to a slight different accommodation of the two scaffolds in the nicotinamide pocket.

**Figure 5:**
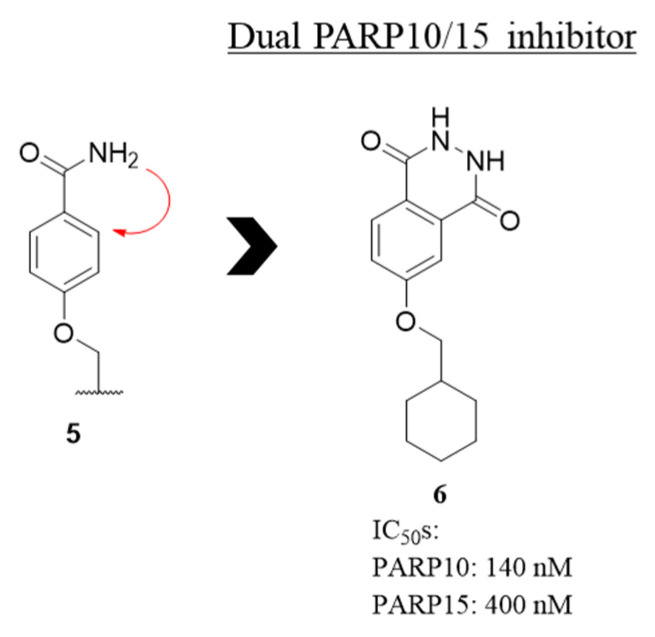
Structure and enzymatic activity of dihydrophthalazindione **6**, derived from structural rigidification of benzamide **5**.

In 2022 Lehtiö et al. reported a set of different phthalazinone-based derivatives. They were designed to improve the potency of some benzamide PARP10 inhibitors (**5**, Figure 5) [95,96], through the rigidification of the amide to give 2,3-dihydrophthalazine-1,4-diones (Figure 5). Differently from the previously mentioned phthalazinone derivatives, the presence of a carbonyl group at C-1 position instead of bulky substituents, caused the complete lack of activity against poly-ARTs (PARP1 and PARP2 IC_50_s > 100 µM), with compound **6** that inhibited only PARP10 and PARP15 with a potency of 140 nM and 400 nM, respectively. This dual PARP inhibitor was not cytotoxic in both HEK293T and HeLa cells, and the activity on PARP10 was confirmed in cellular context (HeLa cells) with EC_50_ value in the low µM range [95].

### 2.2. 4,5-Dihydropyridazin-3(2H)-one

The 4,5-dihydropyridazin-3(2*H*)-one scaffold has gained attention in the recent decades for biological, medicinal and agricultural reasons [97,98,99]. Regarding the medical applications, the pyridazinone nucleus was exploited to develop compounds with antiplatelet, cardiotonic, anti-infective, and antidiabetic properties [100]. 

Concerning the PARP field, while the pyridazinone scaffold was never exploited to obtain poly-ARTs inhibitors, it recently furnished the most potent and specific PARP7 inhibitors. It can be regarded as a simplification of phthalazinones, being the essential part responsible for the three hydrogen bonds that allow it to mimic the nicotinamide. 

**Figure 6:**
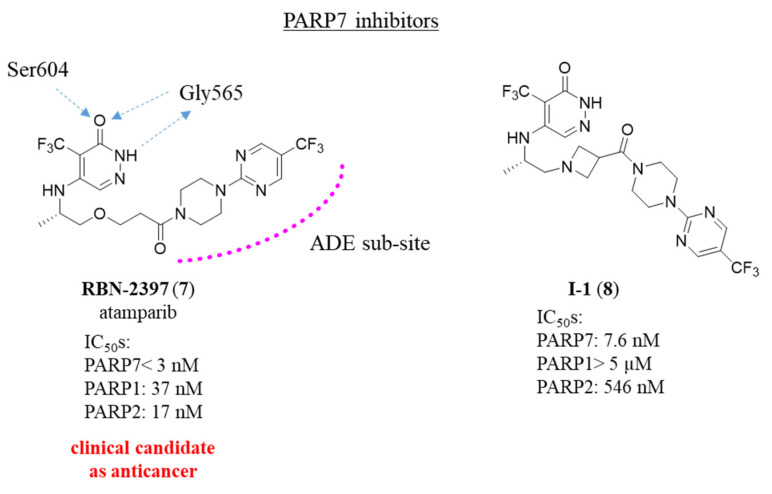
Structure and enzymatic activity of dihydropyridazinones RBN-2397 and I-1. The key interactions between RBN-2397 and the catalytic domain of a mutated PARP12 mimicking PARP7 are also schematically shown.

In 2021, Ribon Therapeutics reported RBN-2397 (**7**, atamparib) (Figure 6) [61], as derived from the structural optimization of an unselective PARPi (whose structure was not disclosed), identified by screening an in-house library. Based on the crystal structure of **7** with a mutated PARP12 mimicking PARP7, the pyridazine group mimics the nicotinamide by interacting with the NI pocket, while the 4-(5-(trifluoromethyl)pyrimidin-2-yl)-piperazine moiety extends towards the ADE site (Figure 6). The compound was very potent in inhibiting PARP7 with IC_50_ value < 3 nM and k_d_ of 0.22 nM measured by SPR. It was very selective with >50-fold over all the whole panel of PARPs. Its activity prevented the PARP7-mediated MARylation of multiple proteins in PARP7-overexpressing SK-MES-1 with IC_50_ = 2 nM, demonstrating also its cell-permeability. 

The specific inhibition of PARP7 by **7** allowed to restore the IFN-I response. This restoration was also responsible for the antitumor effect detected on CT26-tumor bearing mice when treated with the compound. Indeed, the tumor growth was inhibited at doses of >30 mg/Kg for 6 weeks. The activity on PARP7 and, indirectly on IFN-I response, was confirmed by the absence of regression on PARP7 KO tumor-bearing mice and reduced activity in CT26-tumor bearing immunodeficient NOG mice. In addition, KO PARPs 1, 2, and 3 did not affect the activity of **7**, supporting PARP7 as its unique target.

From human xenograft studies, **7** emerged as active in four lung cancer types with complete tumor regression on NCI-H1373. In 2019, it was the first mono-ART inhibitor progressed to clinical trials and it is currently in phase I as anticancer for the treatment of advanced or metastatic solid tumors (NCT04053673) and advanced squamous non-small cell lung carcinoma (NCT05127590). 

A recent study showed also an effect on prostate cancer based on the inhibition of the mono-ADP-ribosylation of androgen receptor (AR). As a consequence, the assembly of the complex formed by AR, PARP9 and E3 ligase DTX3L is inhibited, thereby preventing the AR-mediated transcription. Differently from the immunomodulatory effect observed in the other cancer cell lines, in this case, the antitumor activity is mainly related to the ability of the compound to freeze PARP7 into the nucleus, generating cytostatic and cytotoxic effects and without blocking a specific phase of cell cycle [101].

The interesting anticancer activity of compound **7** demonstrates, for the first time, that anticancer drugs can be also obtained through mechanisms of action that go beyond the classic synthetic lethality or trapping.

In 2022, compound **7** became the starting point for a study performed by Gu et al. [102]. Through a simple bioisosteric replacement of the flexible ethoxy linker of **7** with a rigid azetidine group, compound I-1 (**8**) was created (Figure 6) [102]. It emerged as more specific for PARP7 mainly over poly-ARTs PARP1 and PARP2. Indeed, while the activity of compound **8** on PARP7 was maintained in the same range of **7** (IC_50_ = 7.6 nM), it inhibited PARP1 at 5 µM (versus 37 nM for **7**) and PARP2 at 546 nM (17 nM for **7**). Compound **8** was also endowed with good PK profile in ICR mice through both intravenous and oral administration, also better than that of **7**, tested in parallel, in terms of absorption, exposure, half-life and clearance, and it was non-toxic up 50 µM in normal hepatocytes. However, evaluating its antiproliferative activity in NCI-H1373 cell line, **8** showed a minimal effect when compared with **7**. On the contrary, in CT26 colon carcinoma syngeneic model mouse, oral doses at 25, 50, or 100 mg/kg twice a day determined 67% of tumor growth reduction, while **7** only 30%, probably due to the differences on PK properties. 

### 2.3. Quinazolin-4(3H)-one

Quinazolinone is a ubiquitous structural fragment in medicinal chemistry imparting a broad range of biological activities [103]. This scaffold gave potent compounds active as anticancers such as raltitrexed (thymidylate synthetase inhibitor) currently in use for the treatment of colon-rectal cancer and ispinesib (inhibitor of kinesin spindle protein) currently in phase II clinical trials for the treatment of breast cancer; in parallel, many other compounds have been reported in the literature as anticonvulsant [104], anti-Flu [105], and anti-inflammatory agents [106].

Its wide use is also due to the easy accessibility with a range of cheap and feasible synthetic routes [103]. Quinazolinone is also widespread in the PARPi field with many examples both for poly-ARTs [107,108,109,110] and mono-ARTs [92,94,111]. It perfectly accommodates in the NI site by interacting with highly conserved residues: the NH generates a H-bond with glycine while the carbonyl generates a H-bond with OH of serine; π-π interactions are then performed between the core and tyrosine. 

**Figure 7:**
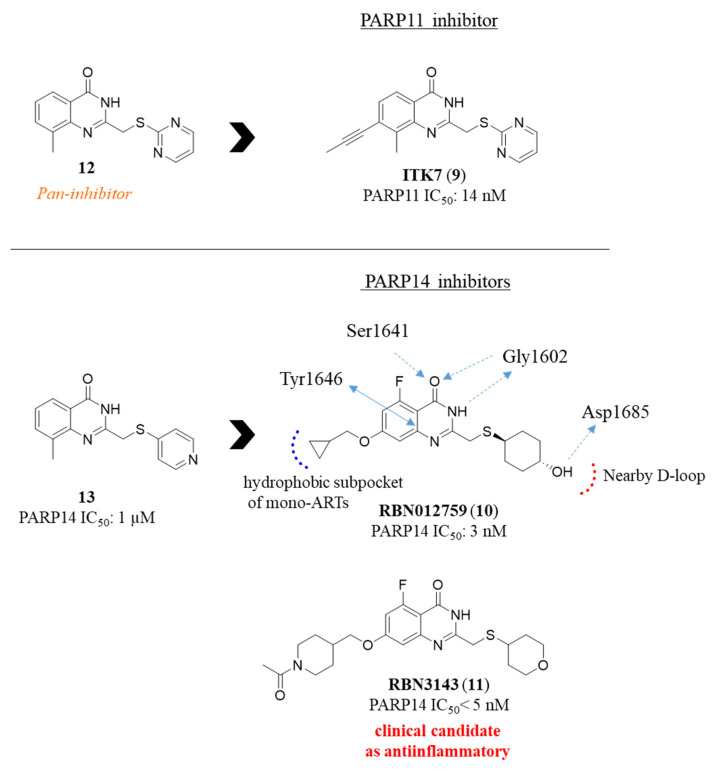
Structure and enzymatic activity of quinazolinones ITK7, RBN012759 and RBN3143. The key interactions between RBN012759 and the catalytic domain of PARP14 are also schematically shown.

To date, three quinazolinone-based compounds are known for their potent and selective activity for a subfamily of mono-ARTs: the PARP11 inhibitor ITK7 (**9**) [92], and the PARP14 inhibitors RBN012759 (**10**) [94] and RBN3143 (**11**) [111] (Figure 7).

In the search for selective mono-ARTs inhibitors, in 2018, Cohen et al. [92] performed a structure-guided design starting from the pan quinazolinone **12** (Figure 7) and trying to take advantages of the main different feature of the catalytic triad of PARPs: a hydrophobic residue that occupies the third position on mono-ARTs in the place of the poly-ARTs glutamate. A small set of compounds were prepared by introducing a small hydrophobic group such as methyl, ethynyl or propynyl at the C-7 position of the quinazolinone scaffold. Similarly to the starting compound **12**, all of the synthesized analogues bear different aromatic rings at the C-2 position that were linked through a methyl sulphide bridge. The evaluation of the compounds on a large panel of poly- and mono-ARTs validated the hypothesis since all the compounds did not recognize poly-ARTs, while showing micromolar activity against some mono-ARTs.

Derivative **9** emerged as the best compound showing potency and high specificity toward PARP11 (Figure 7), with IC_50_ = 14 nM and ≥200-fold selectivity over all the other PARPs. The compound was also able to enter HeLa cells and inhibit intracellular PARP11 activity with EC_50_ = 13 nM, while no PARP1 or PARP10 inhibition was detected. It is characterized by a propynyl group at the C-7 position coupled with a 2-pyrimidine ring that, reaching the less conserved D-loop region, is responsible for specificity for this subfamily of mono-ARTs.

Few years later, following the same strategy, Cohen et al. [93] used the propynyl group coupled with the phthalazinone nucleus, but in this case the designed compound **4** was not selective.

The other quinazolinone-based compounds were both identified by Ribon Therapeutics. In 2021, by screening an in-house PARP-preferring compounds library on PARP14, compound **13** was identified that inhibited the enzyme at 1 µM. Since the thiopyridine at the C-2 position extended toward the D-loop, the authors decided to replace it with a thio-*trans*-cyclohexanol to interact with an Asp1685, a residue uniquely present in PARP14 and PARP15 D-loop. The successive modification involved the shift of the hydrophobic substituent from position 8 to position 7 and its replacement with a cyclopropylmethanol. Analogously to compound **9**, the presence of a hydrophobic substituent at this position prevented interactions with the glutamate residue of poly-ARTs, while improved the affinity for mono-ARTs. In this way, they obtained derivative **10** (Figure 7) that showed a PARP14 IC_50_ = 3 nM and it was 300 to 1000-fold selective over the other PARPs. PARP14 was also inhibited in cellular context in a dose-dependent manner in CFPAC-1 (ductal pancreatic adenocarcinoma cells); PARP14 engagement was also confirmed in in vivo experiments by treating C57BL/6 mice with oral doses of 300 and 500 mg/kg of compound for 7 days, without observing body weight loss. In addition, the compound showed good pharmacokinetic properties with appreciable solubility and permeability coupled with reduced MDR-1 efflux pump affinity.

In a successive study, Cho et al., demonstrated the potential usefulness of **10** also in allergic lung inflammation, in mouse model. In particular, it was observed that the specific PARP14 inhibition reduced all the typical features of allergic response, including IgE levels and mucus formation, after mice allergy induction by *Alternaria alternata* [112].

One year later, the strict analogue **11** (Figure 7) was also disclosed, having tetrahydropyran and *N*-acetyl piperidine, at C-2 and C-7, respectively. It was tested against a PARPs panel, through an enzymatic activity assay or time-resolved fluorescence resonance energy transfer (TR-FRET) probe displacement assay, showing IC_50_ < 5 nM on PARP14, with >300-fold selectivity over all the other PARPs. The compound inhibited also efficiently PARP14-mediated ADP-ribosylation in cellular context with EC_50_ = 3 nM and suppressed the markers when tested in preclinical models of lung skin and gastrointestinal inflammation. Being potent, safe and well tolerated in preclinical models, it was progressed to phase I clinical trials for atopic dermatitis (NCT05215808) [113].

The three quinazolinone-based compounds clearly demonstrate that very minor modifications can strongly modulate the specificity of the compounds. To achieve adequate potency and selectivity the best strategy resides in extending the compounds toward: (i) the third amino acid of the triad granting the selectivity for mono-ARTs over poly-ARTs; (ii) the D-loop that, being not conserved, could allow specificity for a single mono-ARTs. Both goals can be achieved by extending the positions C-7 and C-2 of the scaffold, respectively: hydrophobic substituents are required in C-7 position, while disparate aromatic or nonaromatic substituents can be placed in C-2, with a methyl sulphide spacer that seems preferred.

### 2.4. [1,2,4]Triazolo[3,4-b]benzothiazole

In 2023, the [1,2,4]triazolo[3,4-*b*]benzothiazole (TBT) scaffold has emerged as new chemical entity (NCE), particularly suitable to obtain PARPi [114]. TBT is underexplored in medicinal chemistry with only a few pharmacologically active compounds developed as anti-inflammatory, anticonvulsant or antifungal agents [115,116]. On the other hand, 1,2,4-triazole is a well-known structure found in some drugs with antifungal (fluconazole, itraconazole), anxiolytic (alprazolam) or antiviral (ribavirin) [117] activity along with many other pharmacologically active compounds that continue to appear in the literature [118,119,120]. Many properties make the 1,2,4-triazole a suitable pharmacophore, such as the high stability, its dipole character, solubility, rigidity and ability to generate H-bonds, and its bioisosterism with ester, carboxylic acid and amide. Thanks to its ability to work as nonclassical amide bioisoster, in recent years it was included in the design of TNKS1/2 and PARP1/2 inhibitors [121,122,123], where it mimics the nicotinamide portion of NAD^+^. The inclusion of 1,2,4-triazole on the tricyclic TBT expanded its use giving PARPi with very versatile profile.

The TBT scaffold perfectly fixes on the NI site, with the nitrogens in positions 1 and 2 of the scaffold that mimic the lone pair of the nicotinamide oxygen and generate the H-bonds with glycine and serine. The aromatic portion of the tricycle generated, instead, π-π interactions with the tyrosine residue.

**Figure 8:**
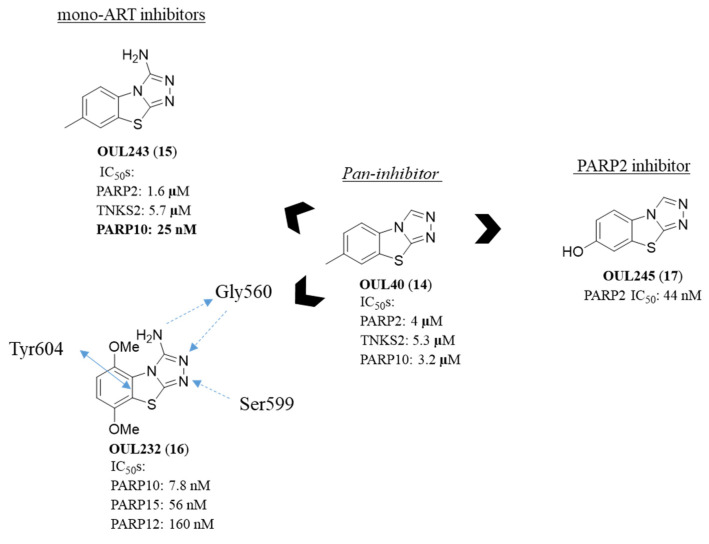
Structure and enzymatic activity of triazolobenzothiazoles **14**–**17**. The key interactions between OUL232 and the catalytic domain of a mutated PARP15 mimicking PARP10 are also schematically shown.

Of note, the TBT derivatives were not rationally designed as nicotinamide mimetic, but they were identified by screening the open chemical repository library of the National Cancer Institute against PARP10, from which OUL40 (**14**) (Figure 8) emerged as able to inhibit this enzyme with IC_50_ = 3.2 µM. It inhibited however other mono- and poly-ARTs in the same µM range. A first optimization campaign, involving the synthesis of about 30 analogues modified both on the benzene ring and the C-3 position, led to compounds selective for one or another PARP subfamily, depending on the substituents pattern. The presence of an amino group in position 3 gave OUL243 (**15**) (Figure 8) that although still active on poly-ARTs, showed a markedly increased potency against PARP10 (IC_50_ = 25 nM), resulting in 10–1000-fold selectivity.

The presence of a couple of methoxy groups instead gave compound OUL232 (**16**) (Figure 8), that with an IC_50_ = 7.8 nM, is the most potent PARP10 inhibitor ever reported. It is also the most potent PARP15 and the first PARP12 inhibitor reported in the literature (IC_50_ = 56 and 160 nM, respectively). Not inhibiting poly-ARTs up to concentrations of 10 µM, **16** emerged as highly selective for mono-ARTs. The co-crystallographic structure of **16** with a mutated PARP15 mimicking PARP10 showed that the 3-amino group works as an anchor point able to generate the same H-bond that the nicotinamide NH_2_ generated with glycine residue, justifying its crucial role in imparting potent activity. The activity of **16** on PARP10 was confirmed in a HeLa cell model (colony formation assay) with EC_50_ = 150 nM. The compound was deprived of cytotoxicity both in HEK293T and HeLa cells and preliminary pharmacokinetic profile suggested good phase I metabolic stability in human liver microsomes, with >99% of unchanged compound and the same good stability was observed in MeOH, PBS buffer and human plasma incubated at 37 °C (>24 h). Derivative **16** exhibited suboptimal GI permeability (PAMPA) with low membrane retention, while the BBB permeability, although suboptimal, was 10-fold higher (P_app_ 0.143 × 10^−6^ cm/s) than that of the clinical PARPi olaparib (P_app_ 0.016 × 10^−6^ cm/s), tested in parallel.

The high versatility of the TBT scaffold in providing selective PARPi was also reflected by 7-hydroxy derivative OUL245 (**17**) (Figure 8), that, in this case, specifically and potently inhibited PARP2 at 44 nM, with even 10-fold selectivity also over the strict parent PARP1. While this specificity was not justified by the co-crystal structure, its selectivity for poly-ARTs over the mono-ARTs derived by the presence of a hydrogen bond between OH group and the catalytic glutamate residue.

## 3. Applications of Specific ARTs Inhibitors

Most of the above reported inhibitors are endowed with the proper profile: (i) that under appropriate optimization could lead to clinical candidates and furnish future drugs; (ii) to be used as chemical probes; (iii) to be manipulated as protein degraders.

### 3.1. Specific Inhibitors as Precious Chemical Probes

Over the past decades, many protocols and techniques have been developed for interrogating the cell biology; for an overview of the main methodologies and techniques used for studying these enzymes, the readers are directed to the following reviews [81,124,125].

In parallel, the availability of high-quality chemical probes is increasingly necessary to permit the visualization of biomolecules in live cells, the cell signaling regulation, the identification of druggable targets and providing the basis for drug development.

To be used as chemical tools, the compounds should satisfy at least some basic criteria, such as permeability, potency and selectivity. If they suffer of poor selectivity or inadequate potency, misleading results could be generated. In the PARP context, the main obstacle is represented by poor selectivity since most of the inhibitors reported to date, including those in therapy, work as NAD^+^ mimetics and, being the NAD^+^ pocket highly conserved among the subfamilies, they usually recognize more than one enzyme.

Working with chemical probes could have the main advantages to inhibit a specific function of the target protein providing a greater control if compared with, for example, the RNA interference that gives a complete protein knockdown, deleting its multiple functions.

The labelling of chemical probes can expand their use. The compounds can be labelled before their use or in situ by exploiting bioorthogonal chemistry.

**Figure 9:**
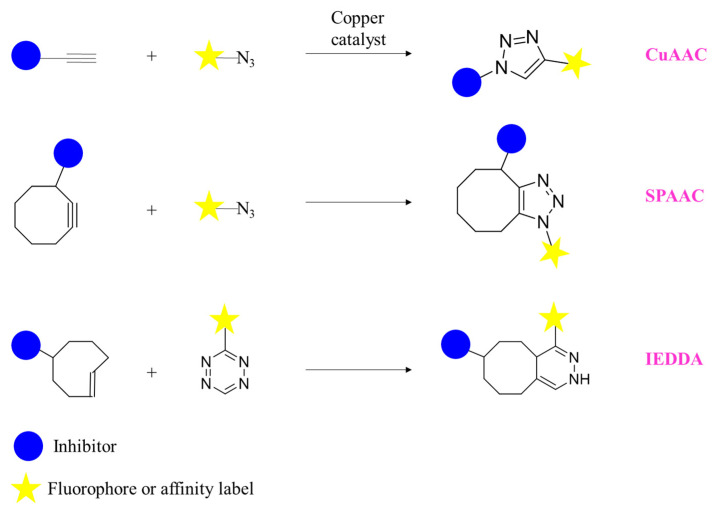
Examples of biorthogonal reactions: CuAAC, SPAAC and IEDDA. SPAAC and IEDDA are copper-free reactions. The functional groups essential for the reactions can be also exchanged between the inhibitor and the fluorophore/affinity label.

The pre-labelling can be carried out by attaching fluorophores allowing to visualize the compound during biological processes; however, these moieties are typically bulky groups that can dramatically affect both the pharmacokinetic profile and the target engagement. Radioactive labelling represents a valid alternative that can be used in various stages of the drug development, form the early phases of the hit discovery up to the clinical trials helping in the determination of pharmacokinetic and pharmacodynamic properties, as well as in the ADME evaluation. Nonetheless, also this approach presents limitations, including the susceptibility of some isotopes to the exchange, especially during in vivo experiments [126]. The bioorthogonal chemistry can overcome some of the mentioned issues. More than 20 different bioorthogonal reactions have been developed, such as copper-assisted azide-alkyne cycloaddition (CuAAC), strain promoted azide alkyne cycloaddition (SPAAC), and inverse electron demand Diels-Alder reaction (IEDDA) (Figure 9). These techniques require a very minor manipulation of the identified chemical probe by introducing a suitable reactive group such as alkyne or azide that react in cellular context with the counterpart (azide or alkyne) in a specific manner (the groups are not present in living system) and in biocompatible conditions.

In the PARPs field, many poly-ARTs inhibitors have been exploited as tools, with most of them represented by radiolabelled compounds, which are also emerging as promising tumor imaging agents [127,128,129,130]. On the contrary, a very few examples of clickable compounds are known [131,132,133,134].

**Figure 10:**
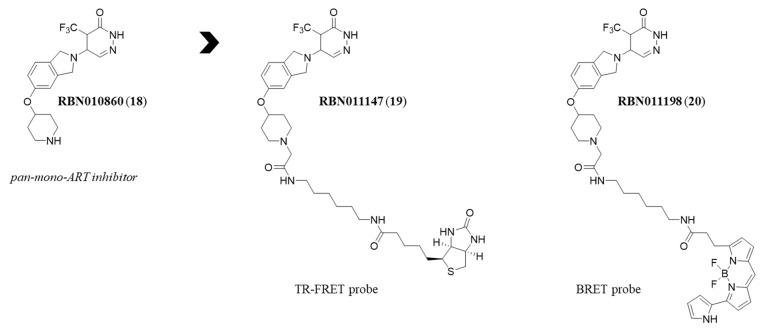
Structure of compound **18** and its derived probes **19** and **20** developed for FRET and BRET, respectively.

Concerning mono-ARTs, the labelling strategy have been exploited in a very few cases. In 2020, using the pyridazinone derivative RBN010860 (**18**) (Figure 10) [135], which is active in the sub-micromolar range against multiple mono-ARTs and also active on poly-ARTs, Ribon Therapeutics prepared two distinct probes in order to develop high-throughput in vitro and cellular biophysical assays. Compound **18** was connected through a flexible chain linked to the piperidine NH, to a biotin moiety or the fluorescent group 590SE, generating derivatives RBN011147 (**19**) and RBN01198 (**20**) (Figure 10), respectively. The biotinylated derivative was useful for the development of a TR-FRET, while the fluorescent probe was used for the bioluminescence resonance energy transfer (BRET). Of note, both the compounds maintained the same potency of the progenitor **18**.

From this work, pyridazinone was confirmed as a very suitable chemical entity to develop mono-ARTs inhibitors. Compared to previous derivatives **7** and **8**, compound **18** is characterized by an isoindoline bicycle instead of the flexible linkers, suggesting that C-5 position can tolerate disparate substituents for further analogues.

Until now, only derivative **4** (Figure 4) was instead used as chemical probe in situ; the presence of the ethynyl group at the C-6 position, besides conferring potent activity against PARP16, made it a clickable compound [93]. Through a CuAAC reaction, the ethynyl group of **4** reacted with tetramethylrhodamine- (TAMRA) azide in cell lysates, allowing the monitoring and then confirmation of the covalent interaction of the inhibitor with PARP16. In addition, the click reaction was also useful for determining the proteome-wide selectivity of derivative **4**, for which PARP16 was confirmed as major target.

Most of the inhibitors described in this review have the appropriate profile of chemical probes to be used as such or after labelling and we expect for these compounds a wide application in the next future.

### 3.2. Protein Degraders as Alternative to Catalytic Inhibitors

**Figure 11:**
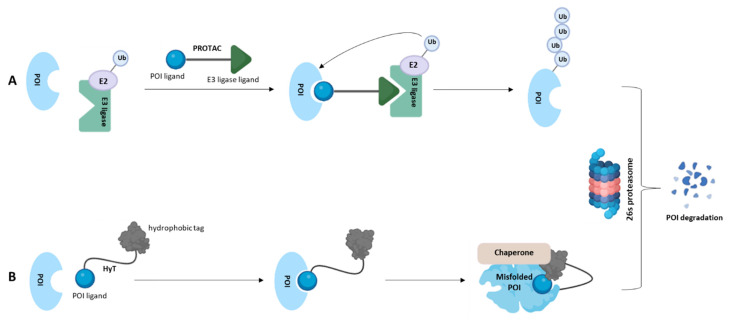
Schematic representations of: (**A**) PROTAC approach: the PROTAC is a heterobifunctional molecule consisting of a POI ligand that recognizes the protein; an E3 ligase ligand that binds the E3 ubiquitin ligase; and a linker. Once the ternary complex POI-PROTAC-E3 ubiquitin ligase is formed, the target protein will be subjected to ubiquitination and then degradation via proteasome. (**B**) Hyt approach: Hyt is a heterobifunctional molecule consisting of a POI ligand that recognizes the protein; a hydrophobic tag that mimics the protein misfolding; and a linker. Once the Hyt recognizes the protein, it recruits cellular chaperone proteins and allows the target degradation via proteasome.

An approach alternative to the catalytic inhibition of the enzyme is represented by its degradation through the chemically mediated targeted protein degradation (TPD) approach. It emerged as a new potential therapeutic modality with possible advantages in terms of side effects, dosing, drug resistance and “undruggable” target modulations [136]. The most representative example of TPD is proteolysis-targeting chimera (PROTAC), which was the founder and still the most exploited [137]. PROTACs are heterobifunctional molecules consisting of a ligand that binds the protein of interest (POI), a moiety (e.g., thalidomide and its derivatives, or nutlin-3) recruiting an E3 ligase (e.g., MDM2, cIAP1, CRBN, VHL), and an appropriate linker joining these two functionalities. PROTACs concomitantly recognize the POI and recruit the E3 ubiquitin ligase which promotes target polyubiquitination and subsequent destruction by the proteasome (Figure 11A).

Although PROTACs have been successfully applied in the degradation of different types of proteins with some that already entered clinical trials [138], this technology still has many disadvantages particularly related to the pharmacokinetic profile of the compounds characterized by high molecular weight, low water solubility, molecular rigidity and low permeability [139,140].

In recent years, the PROTAC approach has been also applied to PARPs field, with the first example of PARP1 PROTAC dating back to 2019 based on niraparib [141]. All the PARP1 PROTACs reported until now were generated from the clinically approved PARPi including rucaparib, veliparib, niraparib and olaparib, with the last one that was the most used ligand that was always extended from the piperazine nitrogen with a variety of linkers. Indeed, from the PARP1 co-crystal structure, it was observed that the cyclopropyl ring, bound to the piperazine nitrogen could be replaced without dramatically affecting the affinity; in addition, it is located close to an opening of the ligand-binding pocket, thus suggesting the possibility to introduce bulky substituents. Concerning the linkers, it was observed that their length, flexibility and composition thinly regulated the PROTAC degradation and selectivity; the typical thalidomide was, instead, the most exploited moiety recruiting the E3 ligase.

**Figure 12:**
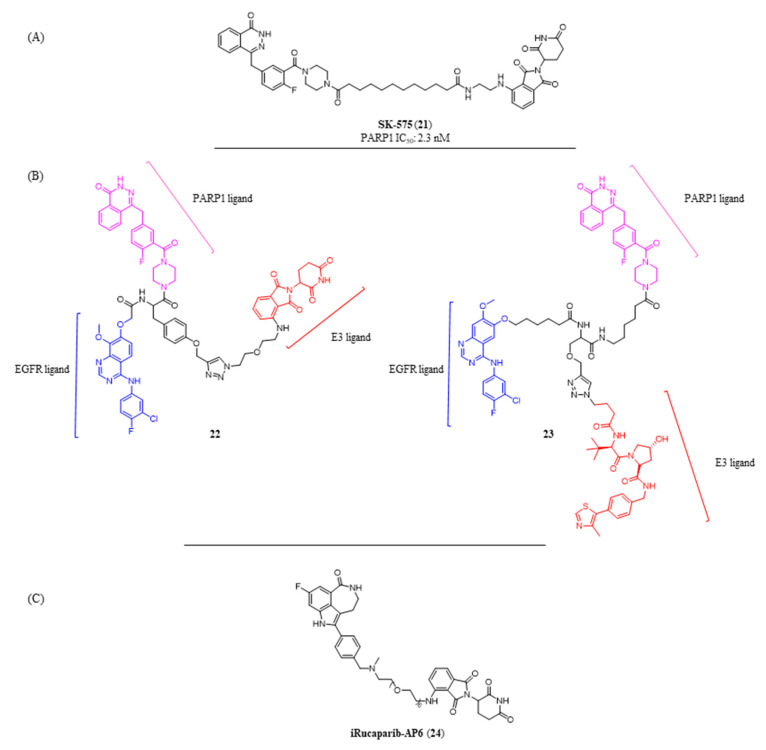
Structures of: (**A**) PROTAC **21**, the most potent PARP1 degrader along with its PARP1 IC_50_; (**B**) dual PROTACs **22** and **23** able to bind and degrade both PARP1 and EGFR; (**C**) PROTAC **23** developed for non-oncological diseases.

All of PARP PROTACs showed good antiproliferative activity, strictly related to the target degradation, with low µM to nM IC_50_ values, comparable or slightly better to that of their parental catalytic inhibitors. Olaparib-based SK-575 (**21**, Figure 12A) [142] emerged as the most potent and efficacious degrader reported to date that achieved DC_50_ values in the picomolar range (228 pM to 578 pM) in a panel of five different cancer cell lines and effectively induced PARP1 degradation in SW620 xenograft tumor tissues in mice with the effect persisting for >24 h.

The PROTACs concept was also expanded by generating dual-targeting PROTACs to simultaneously degrade two different targets. In particular, trifunctional compounds **22** and **23** (Figure 12B) were prepared by connecting a central linker (black) to three different moieties: olaparib (magenta) for the PARP1 engagement, gefitinib (blue) for the interaction with EGFR, and CRBN/VHL E3 ligase ligand (red) [143]. When tested on epidermal carcinoma A431 cell lines, the compounds were able to efficiently degrade both PARP1 and EGFR. However, besides the double targeting they showed reduced drug-like properties that require further optimization.

As known, many of the current PARP1 inhibitors possess both catalytic inhibition and trapping activities. While PARP1 trapping and its cytotoxic effects are desirable in cancer treatment, this could be a limitation when planning to use PARPi in non-oncological conditions [144] such as ischemia-reperfusion injury [145] or neurodegenerative diseases. In these pathologies, PARP1 is hyperactivated and represents the main mechanism of NAD^+^ depletion, responsible for energy crisis and necrosis. The development of a PROTAC could expand the use of PARPi also in these non-oncological diseases. Indeed, the protein degradation determines the same effects of a PARP1 catalytic inhibition, thus allowing protection of the cells from the genotoxic effects determined by NAD^+^ and ATP depletion, but, in parallel, avoids the undesirable DNA damage and cell growth inhibition caused by the trapping. The hypothesis was validated by the PROTAC iRucaparib-AP6 (**24**) (Figure 12C) [146] that, when tested in C2C12 muscle cells and primary cardiomyocytes, potently caused the PARP1 degradation in the low nM range and, differently from the progenitor rucaparib, protected the cells from the death.

**Figure 13:**
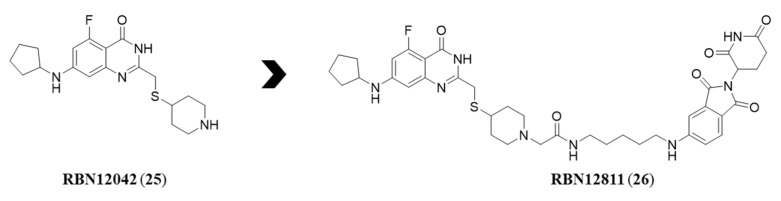
Structures of the first mono-ART PROTAC **26** and its progenitor **25**.

Moving to mono-ARTs field, only the PROTAC developed by Ribon Therapeutics for PARP14 has been reported (Figure 13) [147]. The heterobifunctional compound derived from a quinazolinone-based derivative, RBN01242 (**25**), that specifically and potently inhibited PARP14 with IC_50_ = 18 nM. Compound **25** was functionalized both in position 2 and 7, by using the same strategy already applied for obtaining derivatives **9**–**11**. By manipulating the nitrogen of the piperidine, the compound was elaborated into the PROTAC RBN12811 (**26**) (Figure 13) by introducing a flexible linker to which thalidomide was attached. The inhibitory potency of the derived PROTAC was maintained in the same range of that of **25**, with IC_50_ = 10 nM and still maintained a selectivity > 200-fold over the other PARPs. In addition, when tested on KYSE-270 cells the degradation mechanism mediated by ubiquitin-proteasome pathway was confirmed.

The high potency and selectivity of compound **26** suggest that PARP14 well tolerates bulky substituents extending from the C-2 position of the quinazolinone scaffold, this SAR insight could be further explored in the future.

Along with the very exploited PROTAC approach, alternative TPD strategies have also emerged, such as hydrophobic tagging (HyT), SNIPER, molecular glue, LYTAC, antibody-based PROTAC (AbTAC), glueTAC and autophagy-based PROTAC [148,149]. Until now, only the HyT strategy has been used in the PARPs field, but it is still in its infancy with only one compound, based on olaparib, reported in 2020 [150].

A hydrophobic tagged compound is constituted by a ligand that specifically binds the POI, a hydrophobic and bulky group (e.g., admantane, fluorene, pyrene, tert-butyl carbamate protected arginine) and a variable and flexible linker tethering the two moieties. By exploiting this approach, the protein destabilization is obtained thanks to the hydrophobic fragment that, localizing into the protein surface, mimics target protein misfolding, thus causing the degradation by proteasome or autohpagosome pathway (Figure 11B). Compared to PROTAC, this strategy offers several advantages including better physiochemical and pharmacokinetic properties due to a lower molecular weight and the elimination of the risk of teratogenic side effects from thalidomide-derived CRBN ligands.

**Figure 14:**
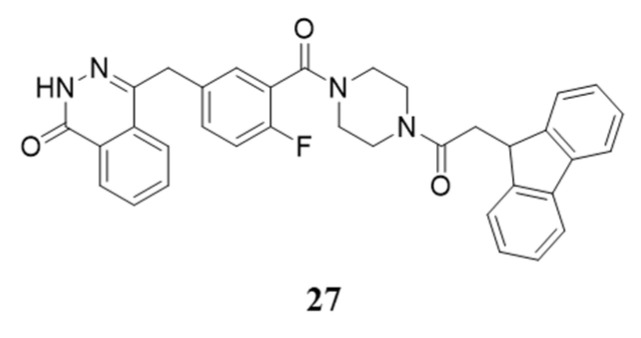
Structure of the first Hyt for PARP1, compound **27**.

Olaparib was, again, selected as protein ligand moiety, while fluorene emerged as the best hydrophobic tag, which was linked to warhead with a two-carbon spacer. Being responsible for the targeted protein misfolding, the obtained compound **27** (Figure 14) efficiently promoted PARP1 degradation via the proteasome-dependent pathway. Compared to olaparib, **27** showed a better antitumor activity on triple negative breast cancer, also due to its ability to cause the unfolded protein response (UPR) activation and encourage apoptosis and autophagy [150].

## 4. Conclusions and Future Perspectives

In the cancer precision medicine field, PARPi have represented an important step forward since 2014, when olaparib was approved followed by three other PARP1/2 inhibitors used as single agents or in combination for the treatment of BRCA1/2 deficient tumours, revolutionizing for example the handling of advanced ovarian cancer. They work mainly based on a synthetically lethal effect, but additional mechanisms justify their advantageous application, including a contribution by pan PARPs inhibition.

With respect to PARP1/2, the most abundant and best studied members of the PARP family that work as poly-ADP-ribosyltransferases, the studies on mono-ARTs, to which 14 out 17 PARPs belong, date back to just over 20 years ago [151]. Since then, the research around these enzymes has rapidly expanded, clarifying their physiopathological functions and highlighting their potential as drug targets. Playing key roles in DNA damage, apoptosis and cell cycle regulation, they have been already linked to diseases such as cancer and inflammation. Other disparate pathological conditions in which these enzymes are involved continue to emerge [124], that however need for specific mono-ARTs inhibitors to be fully dissected. Most important, specific mono-ARTs inhibitors could represent next-generation drugs. Indeed, even if the polypharmacology could be beneficial in some cases, the promiscuous interactions with the multiple targets, including multiple PARPs subfamilies, could be harmful and responsible for serious off-targets effects. This has been already confirmed for poly-ARTs inhibitors, where the new drugs are moving toward specific PARP1 inhibitors.

Unfortunately, mono-ARTs inhibitors are still scarce mostly because of the high similarity of the catalytic domain where almost all of the inhibitors work. For example, the classical nicotinamide bioisoster benzamide has been widely used to design both poly-ARTs and mono-ARTs inhibitors [95,96,152,153,154,155] but none of them emerged as particularly specific. The most specific and potent mono-ARTs inhibitors were collected in this review underlining some privileged scaffolds: monocycle pyridazinone, bicycles quinazolinone and phthalazinone, and tricycle triazolobenzothiazole.

Phthalazinone and its quinazolinone isoster, already largely exploited for poly-ARTs inhibition, were confirmed as suitable also for achieving mono-ARTs inhibitors, such as the phthalazinone derivative KMR-206 (**1**), selective for PARP7, and quinazolinones derivatives: RBN012759 (**10**) and RBN3143 (**11**), selective for PARP14, and ITK7 (**9**), selective for PARP11.

The other two privileged scaffolds are pyridazinone and TBT that, being not used before even for obtaining poly-ARTs inhibitors, have emerged as NCEs within PARPi field. In particular, pyridazinone-based compounds RBN-2397 (**7**) and I-1 (**8**) showed a specificity for PARP7, while TBT was more versatile, giving derivatives specific for one or another subfamily. Based on the substitution pattern, compounds such as OUL232 (**16**), which is selective for PARP10, or OUL245 (**17**), that showed a preference for PARP2, were obtained.

All of the compounds reported in this review represent valid chemical probes to fully dissect the physiophatological roles of mono-ARTs enzymes, and, most important, for some of them the pharmacological effect of their inhibition has been already successfully investigated.

The clear therapeutic potential of mono-ARTs inhibitors is also confirmed by the increasing interest of pharmaceutical industries, such as AstraZeneca or Ribon Therapeutics. Ribon Therapeutics efforts have already led to RBN-2397 (**7**) and RBN3143 (**11**), which entered clinical trials as anticancer and anti-inflammatory, respectively, confirming mono-ARTs as valid drug targets, not only in cancer where they represent a new weapon for the treatment of cancers without BRCA mutations, but also in other diseases, expanding the horizon of PARPi-based therapies.

In summary, from this review it emerged that (i) few scaffolds are able to give specific inhibition, mostly because they came by screening PARPs-preferred proprietary libraries; (ii) the same scaffold furnishes compounds with different selectivity profiles, under minor manipulation; (iii) with rare exceptions, no clear SAR emerged.

Thus, starting from the above catalytic inhibitors, we invite the medicinal chemists to undertake hit-to-lead optimization campaigns to achieve specific compounds for each of the mono-ARTs subfamily, both as precious chemical probes and future drugs with many potential therapeutic applications. A few alternative approaches that are almost underexplored in the mono-ARTs field are protein degradation, as mentioned before, or macrodomain inhibition, where a few examples of inhibitors were already developed for PARP14 [156,157], but could be beneficial also for PARP9 and 15, equally characterized by macrodomains.

## Data Availability

Not applicable.

## Abbreviations

ADE	adenine ribose
ARTs	ADP-ribosyltransferase
ARTDs	diphtheria-toxin-like ADP-ribosyltransferases
BRCA	breast cancer gene
BRET	bioluminescence resonance energy transfer
CuAAC	copper assisted azide alkyne cycloaddition
ERα	estrogen receptor
HDAC	histone deacetylase
Hyt	hydrophobic tagging
HR	homologous recombination
IEDDA	inverse electron demand Dies-Alder reaction
NAD^+^	*β*-nicotinamide adenine dinucleotide
NCE	new chemical entity
NHEJ	non-homologous end joining
NI	nicotinamide
PARPs	poly-ADP-ribose polymerases
PARPi	PARP inhibitors
PCNA	proliferating cell nuclear antigen
POI	protein of interest
PROTAC	proteolysis-targeting chimera
PTMs	post-translational modifications
SPAAC	strain promoted azide alkyne cycloaddition
SSBs	single strand breaks
TBT	[1,2,4]triazolo[3,4-*b*]benzothiazole
TNKS	tankyrase
TPD	targeted protein degradation
TR-FRET	time-resolved fluorescence resonance energy transfer
β-TrCP	β-transducin-repeat containing protein
UPR	unfolded protein response
